# Unveiling Plant-Based Pectins: Exploring the Interplay of Direct Effects, Fermentation, and Technological Applications in Clinical Research with a Focus on the Chemical Structure

**DOI:** 10.3390/plants12142750

**Published:** 2023-07-24

**Authors:** Lucas de Freitas Pedrosa, Karen Rebouças Nascimento, Caroline Giacomelli Soares, Débora Preceliano de Oliveira, Paul de Vos, João Paulo Fabi

**Affiliations:** 1 Department of Food Science and Experimental Nutrition, School of Pharmaceutical Sciences, University of São Paulo, São Paulo 05508-000, SP, Brazil; 2 Department of Pathology and Medical Biology, University Medical Center Groningen, University of Groningen, 9713 GZ Groningen, The Netherlands; 3 Food and Nutrition Research Center (NAPAN), University of São Paulo, São Paulo 05508-000, SP, Brazil; 4 Food Research Center (FoRC), CEPID-FAPESP (Research, Innovation and Dissemination Centers, São Paulo Research Foundation), São Paulo 05508-080, SP, Brazil

**Keywords:** pectin, functional properties, fermentation, human health, dietary fiber, technological application

## Abstract

Pectin, a plant-derived polysaccharide, possesses immense technological and biological application value. Several variables influence pectin’s physicochemical aspects, resulting in different fermentations, interactions with receptors, and other functional properties. Some of those variables are molecular weight, degree of methylation and blockiness, and monosaccharide composition. Cancer cell cytotoxicity, important fermentation-related byproducts, immunomodulation, and technological application were found in cell culture, animal models, and preclinical and clinical assessments. One of the greater extents of recent pectin technological usage involves nanoencapsulation methods for many different compounds, ranging from chemotherapy and immunotherapy to natural extracts from fruits and other sources. Structural modification (modified pectin) is also utilized to enhance the use of dietary fiber. Although pectin is already recognized as a component of significant importance, there is still a need for a comprehensive review that delves into its intricate relationships with biological effects, which depend on the source and structure of pectin. This review covers all levels of clinical research, including cell culture, animal studies, and clinical trials, to understand how the plant source and pectin structures influence the biological effects in humans and some technological applications of pectin regarding human health.

## 1. Introduction

To be well established in the environment, plants carry some mechanisms to guarantee their rigidity, stability, and flexibility [1]. A specific region, named the middle lamella, results from cytokinesis in the first steps of plant development. Soluble proteins and peptides compose this region, together with cellulosic material (either cellulose microfibrils or hemicellulose) and one central mediator between fruit pulp’s rigid and flexible structures: pectin [2].

Pectin is a heteropolysaccharide, which means it is composed of a variation of monosaccharide residues [3]. As early as 1825, the main constituent of pectin was isolated and explored: galacturonic acid (GalA) [4]. Later on, many other residues were identified to be intercalated or present in side-chain molecules throughout the pectic linear chain. Major functions in plants revolve around maintaining cell-to-cell adhesion, as well as influencing cell growth and extension [5,6]. Pectins can have significant molecular sizes in their native form, but during ripening, pectin is hydrolyzed by polygalacturonases and other enzymes that act on its side chains, resulting in pulp/peel softening and a de-complexation of the targeted molecules [5,7].

Pectic polysaccharides contribute to better human health. Initially, pectins were considered dietary fibers and potential prebiotics that modulate a healthier and more diverse intestinal microbiota. Pectin ingestion results in an enhancement in the production of metabolites such as indols and short-chain fatty acids (SCFAs), which are beneficial to the local intestinal environment resulting in positive systemic effects. In addition, pectin can exert Gal-3 protein inhibition, Toll-like receptor (TLR) interaction, apoptosis induction, and other functional biological properties.

The industrial value of pectin is immense. They can be applied as natural thickeners, stabilizers, and even emulsifiers [8,9,10]. However, a breakthrough technological application of pectins is related to human health. Matrixes involving pectin with other molecules, such as synthetic polymers, chitosan, proteins, and other polysaccharides, have been assembled as micro- and nanoparticles that were able to carry functional components, such as curcumin and polyphenols and deliver them safely after digestion to sites for intestinal absorption, passing intact through the stomach with a lower pH, digestive enzymes, and shear forces in the gastrointestinal tract [11,12,13,14]. We will explain in the following sections how the plant source and pectin structures can influence the biological effects in humans, covering all levels of clinical research, including cell culture, animal studies, and clinical trials. Moreover, some technological applications of pectins regarding human health are also addressed since exploring different application techniques focusing on human health promotion is one of the goals of this review.

## 2. Chemical Structure

### 2.1. Backbone Structure of Pectin and Ramifications

**Figure 1:**
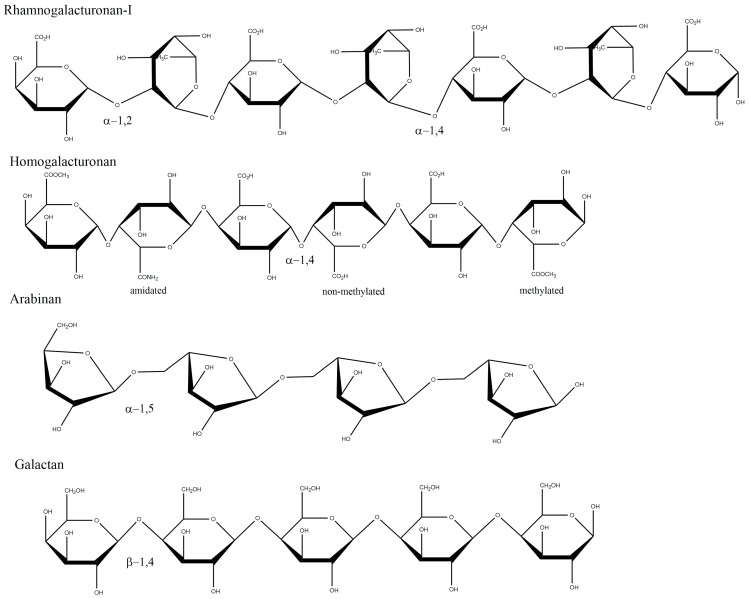
Schematic representation of the most common pectin structures and their respective glycosidic linkages and common substitutions, such as amidation and methylation. Drawings were made using the ChemDraw software.

Pectin’s structural complexity is high and can be composed of many different sugar units. The main part, however, is composed of “smooth”, non-ramified regions containing repeated α-1,4-D-galactopyranuronic acid (GalpA) units, named homogalacturonans (HG). As a carboxylic acid, mainly methyl groups can esterify it at *O*-6, but acetyl groups could be esterified at O-2, O-3, or both [15]. Occasionally, GalpA units can be intercalated with α-1,2-L-rhamnopyranoside (Rhap). This region is called rhamnogalacturonan (RG) and can be ramified in the Rhap residues, depending on the plant source and extraction method. Most commonly, the monosaccharides present in those side chains are α-1,5-L-arabinofuranoside (Araf) and β-1,4-D-galactopyranoside (Galp), forming structures identified as arabinans (linear Ara*f* chains), galactans (linear Gal*p* chains), and arabinogalactans (linear Gal*p* chain with occasional linked Ara*f* in *O*-3 or *O*-6 residues), delineating the Type-I RG [1,3,16,17,18] (Figure 1). The most variable and complex structure in pectin is the Type-II RG, which consists of the same linear conformation as HG but with wide side chains comprising 12 different monosaccharides (some of them rare sugars), such as D-Xylose, D-apiose ((3R,4R)-4-(Hydroxymethyl)tetrahydro-2,3,4-furantriol), L-aceric acid (3-C-Carboxy-5-deoxy-L-xylofuranose), and Kdo (2-Keto-3-Deoxy-D-Mannooctanoic Acid [19]. All those different fragments have been described as having potential health effects, which enhances the curiosity in finding different forms of isolating, improving, or modifying these pectin structures [3,18,20]. 

### 2.2. Degree of Methyl-Esterification and Degree of Blockiness

The degree of methyl-esterification (DM) encompasses the proportion of GalpA units that have been esterified at the carboxylic groups of *O*-6. This value can be obtained by different techniques such as Fourier-transform infrared spectroscopy (FT-IR), nuclear magnetic resonance (NMR), and chemical titration of free galacturonic acid units, and it is used to measure and identify the best type of application of a determined pectin sample [21,22,23]. High-DM pectins can be utilized to form powerful gels without the need for divalent ions [24]. The DM is also fundamental for establishing the components of a potential nanoparticle capsule, where different accessory molecules, such as peptides or other polymers, can enhance or impair stability. The amount of “unmethylated” GalpA units will determine the net charge throughout the molecule, establishing potential binding sites throughout the molecule, which can lead to higher electrostatic interactions with a determined matrix, also depending on pH and pKa values [25,26]. Hydrophobic interactions between methyl and acetyl groups from pectin and hydrophobic amino acids from proteins can further enhance stability. Furthermore, ammonia or primary amine groups, in a process named amidation, can substitute the hydroxyl group linked to C-6, and it can be used to improve the limitation of solubility, changing the structure of Gal*p*A residues into amides [13,27]. Low-DM pectins can be used to coat nanoparticles with a stronger negative charge, protecting them against thermal treatment and pH changes in the environment [26]. 

Not only the proportion of methylation but also the frequency and distribution of methylation are critical factors for the biological effects [17]. The distribution of methylation is called the degree of blockiness (DB). A high DB consists of a block-wise distribution, with a sequence of non-methylated units intercalated with another methyl-esterified sequence [28]. This type of conformation results in locally charged density parts, which can favor anchorage bindings. On the other hand, lower DB indicates high randomness of methyl group allocations, with an absence of those “high-binding prone” regions of interest [26]. 

### 2.3. Molecular Weight and Structure Modification Procedures

Pectins are very large and complex biomolecules. Due to its function in the cell wall, it can reach very long chains, resulting in a high molecular weight when in its native form (without modification) [29]. Some fruits, e.g., papayas, inherently have a high metabolic profile, where their metabolic activity is greatly boosted, targeting the upregulation of energy-producing enzymes, achieving a fast-ripening process. This is deeply associated with pectin hydrolysis during ripening. Polygalacturonases (PGs), pectate-lyases (PL), pectinesterases (PME), arabinofuranosidases (AFs), galactosidases (GAL), and other enzymes, for example, are responsible for the massive reduction in molecular weight observed in papaya fruits from one to five days after harvesting [7,30]. 

However, other fruits, such as passion fruit and citrus, do not have a great alteration in pectin abundance or structure throughout ripening. Nevertheless, it is possible to reduce the molecular weight of those native pectins through artificial methods, such as exogenous enzyme usage, organic or mineral acid extraction, heat and pressure application, ultrasonic degradation, and many others [31,32,33,34,35]. 

Conventionally, de-esterification by both enzymes and alkaline treatments are conventional ways to modify pectin samples, enhancing negative charge-related properties. Acid and alkali treatment, concurrent with mild to high temperatures (between 60 and 90 °C), are the most utilized methods to depolymerize the high-weighted pectic chains, resulting in smaller fragments [36,37]. These methods, however, do generate great amounts of pollutants, being far from ecological approaches when prospecting high-volume production and commercialization. Alternatively, the use of organic acids can achieve similar results in chain reduction, such as tartaric, malic, and citric acids, reaching final molecules with an average molecular weight (Mw) between 26 and 110 kDa [38]. Other methods have also been employed, such as enzymatic hydrolysis with polygalacturonases, usually with arabinofuranosidase, galactanase, and methylesterases [39,40]. The high cost and time, an inherent characteristic of this process, can preclude their commercial use. 

More green-labeled modifications have been targeted, considering the above-described issues [41]. Ultrasound-assisted, for example, is a type of modification that has a variable temperature, time, and frequency, which propagates waves throughout the liquid system, resulting in glycosidic linkage hydrolysis [42,43]. This can also be associated with other methods to improve efficiency and time, such as including pectinases or using hydrogen peroxide systems. On the latter, the Hu et al. (2019) group has optimized a system with sodium hydrocarbonate (NaHCO_3_) and hydrogen peroxide (H_2_O_2_) alongside ultrasonic treatment, achieving a molecular weight reduction of thirty times compared to the native form [44], exemplifying how powerful the technique can be with this objective. High-temperature and high-pressure methods, such as autoclaving or dynamic microfluidization, were also successful in reducing Mw, generating 24 kDa and oligosaccharide fragments, respectively [45,46].

Those alternative extractions and modifications, aligned with the excess of fruits and vegetable byproducts from the industry, promote positive expectancy in pectin extraction, modification, and commercialization. Some of the modification processes and their resulting structures are listed in Table 1.

### 2.4. Low Molecular Weight Pectins for High-End Applications

Low molecular weight pectins exhibit improved biological effects compared to high molecular weight pectins due to several factors. Low molecular weight pectins have smaller sizes and increased water solubility, which enhances their ability to be absorbed and utilized by the body [47]. The higher degree of methylation in low molecular weight pectins results in improved interaction with cellular surfaces, leading to enhanced biological activity [48]. Furthermore, the higher degree of esterification in low molecular weight pectins increases their resistance to degradation and enhances their biological stability [49]. Additionally, some studies have indicated that low molecular weight pectins can interact with specific human cellular receptors, leading to improved biological effects [3,50].

## 3. *In Vitro* Exploration of Pectin Biological Effects

Depending on the study model used, the inferences about pectin effects can be attributed to its structure and epitopes of interest for direct protein/receptor interactions, solubility and fermentation pattern, immune modulation, or a mix of those different effects. It will be discussed in the following sections under other subsections to avoid ambiguity and confusion.

### 3.1. Cytotoxicity Effects of Native and Modified Pectins on Cancer Cells

It is already known that pectins are plant-derived bioactive food polysaccharides with potential cytotoxic properties [51]. Several in vitro studies regarding those properties have been published over the years. Different origins were used for the extraction of pectins, such as papayas [52], sweet potatoes [53], apples [54], citrus [49], olives [55], and sugar beet [56]. Those studies have targeted a wide variety of cancer models, such as breast cancer, pancreatic cancer, urinary bladder cancer, prostate cancer, and colon cancer [51]. 

Metastasis inhibition and cancer cell DNA damage are some of the studied effects of this polysaccharide [47]. However, studies are showing that the biological effects of pectins are directly linked to their chemical structure. For example, pectins extracted from sugar beets without any modification did not show a significant decrease in the viability of colon cancer cells; however, alkali treatment increased this effect [56]. The authors suggested that this effect is because the alkali treatment changed the chemical composition of pectins, increasing the ratio of RG-I to HG content and decreasing the degree of esterification.

The ripening of fruits is a natural cause for changing pectins’ structure. This phenomenon has been studied in climacteric fruits such as papaya (*Carica papaya*), where this specific ripening provides noticeable changes in its pulp structure [57]. The papaya pulp’s cell wall is composed of large cells where the cellulose and xyloglucan framework are involved in a pectin matrix. As the ripening proceeds, the pulp softens by the action of specific enzymes, including endo-polygalacturonases and exo-galactosidases, which degrade the cell wall’s pectin, depolymerize the pectic polysaccharides, and enhance the soluble sugar content in those fruits, in a high metabolic state. HG and RG-I fragments obtained by the action of enzymes on ripening have been explored for inducing cancer cell detachment, which could mean potential cytotoxic activity [52]. 

However, different post-harvest ripening points of papaya’s pectin demonstrated distinct results in a study with two different human colon cancer cell lines, HCT116 and HT29, and a prostate cancer cell line, PC3 [52]. The sample extracted from the intermediate ripening phase (3 days after harvest—3DAH) decreased cell viability and induced necroptosis in these three cancer cell lines. This intermediate ripening point presented smaller chains of pectin with reduced molecular weight (102 ± 5 kDa), which has been suggested as one plausible pivotal factor for a higher modulation of cancer cell survival. Another structural difference between the samples is that this 3DAH period yielded smaller HG chains, smaller RG-I side groups, and AGII associated with RG-I. These results demonstrated that the enzymes of natural ripening acted in different proportions on cell wall pectin over time and alongside many others, opening the discussion about exogenous modifications to standardize the structure and molecular weight of interest.

Low molecular weight citrus pectin (LCP) was used to reduce the proliferation of two different human cancer cell lines, AGS gastric cancer and SW-480 colorectal cancer [58]. LCP contains abundant galactans, which are believed to antagonize galectin-3 (GAL-3), a pro-metastatic protein that has a critical role in the cancer cell profile. LCP treatment reduced cell viability and decreased GAL-3 levels in both cancer cell lines in vitro [58]. However, many others have contested the ability of pectins and other high-weight polymers to inhibit Gal-3 [59].

When pectin with a variable molecular size is tested, different effects can be observed. Two different human colon cancer cell lines, HCT116 and HT29, and a prostate cancer cell line, PC3, were treated with modified citrus pectin (MCP) produced by thermal treatment and fractionated in different molecular sizes, generating different fragments: MCP30 (higher than 30 kDa), MCP30/10 (between 30 and 10 kDa), MCP10/3 (between 10 and 3 kDa), and MCP3 (smaller than 3 kDa) [49]. MCP30/10 had more esterified HG, while type I arabinogalactans (AGI) were more abundant in MCP10/3 than MCP and MCP30. Both MCP30/10 and MCP10/3 had lower amounts of rhamnogalacturonans (RG-I). The smaller molecular size MCP3 had less esterified HG and the lowest amounts of AGI and RG-I. The enrichment of AGI and more esterified HG oligomers in MCP fraction structures showed enhanced cytotoxic effects. All MCP fractions exhibited reduced cell viability in all cell lines, but there is a distinct effect on cell death in different cancer cell lines because this study resulted in divergent effects on proliferation, migration, and aggregation on each cancer cell line with each MCP fraction treatment, showing that these effects are structure-, size-, and cell line-dependent [49]. 

### 3.2. Gut Microbiota Affects Pectin Fermentation

Although not digested by the human gastrointestinal tract, pectin is entirely fermented by the gut microbiota, serving as a healthy substrate for gut microorganisms [60]. Several in vitro gut models have been used to study the impact of pectin on gut microbiota, and exciting data presented in some studies demonstrate that the chemical structure of pectins directly affects their fermentation degree based on their complex molecular structure [60]. Five structural characteristics were identified as the most important in the interaction between gut bacteria and pectins: the amidated groups, the DM, the distribution of HG and RG fractions, the composition of neutral sugars, and the degree of branching [61]. Nevertheless, it is important to realize that different microorganisms exhibit specific preferences for defined substrates [60].

The DM will influence the location at which the pectin is fermented by the gut microbiota. It may have a moderate effect on gut microbiota, depending on the initial population [61]. Some studies illustrate that low-methoxy (LM) pectin fermented faster than high-methoxy (HM) pectin [62,63,64], while other authors also showed that HM pectin fermentation produced more SCFA (short-chain fatty acid) than LM pectin [61]. Lower DM pectins are completely fermented in the upper part of the gastrointestinal tract, while higher DM is fermented to a much higher degree in more distal regions [65]. Another study demonstrated that DM has not influenced the pectin fermentation process by the gut microbiota [65]. However, the fermentation of LM and HM pectins is influenced by initial gut bacteria species that affect fermentation kinetics [60], for example, *F. prausnitzii,* which prefers HM as a substrate compared with LM pectins.

In an in vitro model, *Lactobacilli*, *Bacteroides*, and *Prevotella* populations increased in the gut due to pectin interaction and metabolism [17]. Bacterial strains, such as *Prevotella copri*, *Bacteroides* spp., *Bifidobacterium*, *Coprococcus*, *Ruminococcus*, *Dorea*, *Blautia*, *Oscillospira*, *Sutterella*, *Faecalibacterium prausnitzii*, and *Christensenellaceae* oscillated depending on the type of pectin, corroborating the fact that the biological effects of pectins are chemical-structure dependent.

Therefore, microbial preference over a pectic substrate and metabolite production capability according to the pectin structure can generate higher diversity and environmental health stimuli in the intestinal microbiota through both indirect modulations of immune and epithelial cells, mucin production, lower barrier disruption, and others [61]. Five structural characteristics were identified as the most important in the interaction with pectins: the amide groups, the DM of HG regions, the distribution of HG and RG fractions, the composition of neutral sugars, and the degree of branching [61].

### 3.3. Pectin Beneficial Immunomodulation

Pectin has been shown to interact with cells of the immune barrier in the small intestine [17], mainly through interaction with pattern recognition receptors (PRR) [66]. PRRs can be divided into toll-like receptors (TLR) and nucleotide-binding oligomerization domain (NOD)-like receptors (NLR). TLRs are an important family of receptors and the core structure of the innate immune response, which means that they are the first defense mechanism activated upon damage or pathogen invasion, allowing the response of the adaptive immune system to initiate an antigen-specific response [67,68]. 

Pectin action is dependent on its chemical structure, which was demonstrated by Prado et al. (2020) [66] in a study with papaya pectin at different ripening stages interacting with different TLRs (toll-like receptors) and NOD1 and 2 (nucleotide-binding oligomerization domains). The ripening process changes the structure of papaya’s pectin, resulting in pectins with a higher DM and smaller galactan chains on ripe fruits [66]. The results supported the initial idea of structure-dependent properties of pectin in immunity since all the TLR and NOD receptors were activated by ripe papaya pectin, while only TLR2, 4, and 5 were activated by unripe papaya pectin, whereas TLR3 and 9 were inhibited.

Another study correlated pectin oligosaccharides (POS), a degraded form of polysaccharides present in pectin, with oxidative and inflammation-activated pathways, such as NF-κB, ATP-activated protein kinase (AMPK), and nuclear factor erythroid-2-related factor-2 (Nrf2) [69]. POS affects antioxidant and anti-inflammatory pathways in different ways because of the intrinsic diversity of its structure, which can be standardized with chemical modification. Despite that, POS has the potential to control inflammatory and oxidative stress [69]. 

Pectin has been shown to interact with the cells of the immune barrier in the small intestine [17]. Pectins have a direct interaction with the cells of the gastrointestinal immune barrier, which is composed of multiple layers, containing a mucus layer, a layer of epithelial cells and intraepithelial lymphocytes, and the lamina propria, which is the homing site of innate immune cells [17]. Pectins can affect these layers at different levels. In the mucus layer, low DM pectins can strengthen it by influencing goblet cells, and very high DM pectins can have mucoadhesive effects, forming hydrogen bonds with mucins. In the epithelial layer, pectins can maintain a strong junction structure and protect the integrity against barrier-disrupting agents. In the lamina propria, pectins can be transported by the microfold cells and interact directly with the immune cells, thus influencing their responses. However, there are controversial results about the interaction between different chemical structures of pectin and innate immune cells, demonstrating that pectin can enhance or inhibit the response of immune cells [17,70,71]. The main findings regarding pectin in vitro effects are detailed below in Table 2.

## 4. *In Vivo* Exploration of Pectin Biological Effects

**Figure 2:**
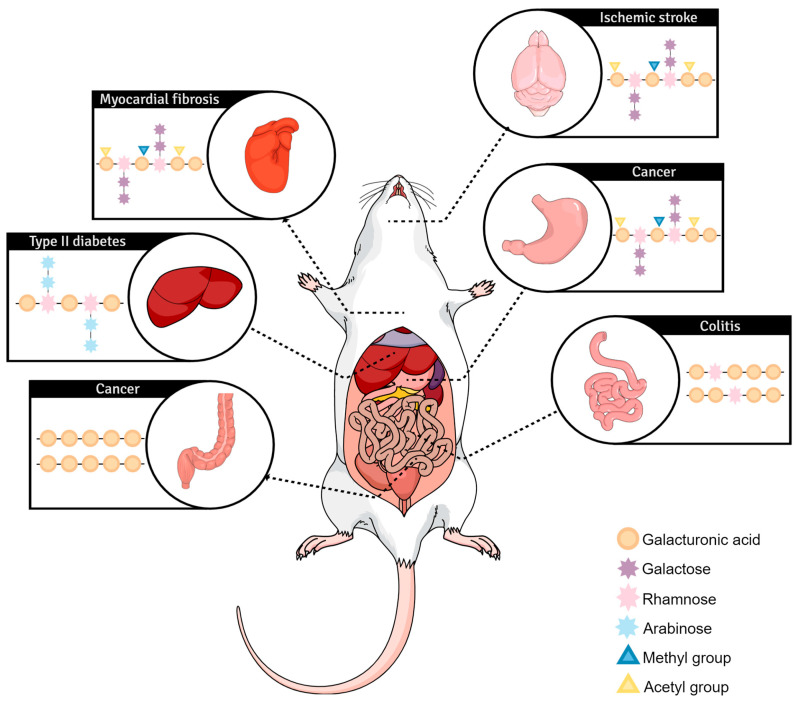
Schematic depiction of variable pectin molecules and their respective region of higher biological activity.

While in vitro studies provide valuable insights into the cellular and molecular mechanisms underlying the effects of pectin in a controlled laboratory environment, in vivo studies provide a more accurate representation of the safety and efficacy of a substance or treatment on a complex, dynamic living organism. They also provide insights into the mechanisms of action and potential side effects, accounting for the complex interactions between the pectin, gut microbiota, and the host’s immune system, as well as the administration route and bioavailability [1]. In vivo studies can help validate the findings of in vitro studies and provide information about the physiological relevance of the effects observed in vitro. Studies have demonstrated the beneficial effects of native and modified pectin on various health outcomes and different organs (Figure 2), including anti-cancer activity, weight management, lipid metabolism, and glucose control.

In general, the most studied dietary fiber is modified citrus pectin (MCP), a type of pectin that has been chemically altered to decrease molecular complexity while increasing its solubility and bioavailability, as already discussed [49]. Cui et al. [77] showed a neuroprotective effect of MCP on ischemic stroke in mice by inhibiting the protein galectin-3. The same mechanism was observed in a mouse model of myocardial fibrosis, although modified rhubarb pectin (EMRP) was found to be the most potent galectin-3 inhibitor when compared to MCP. Screening analysis revealed that EMRP was abundant in galacturonic acid with the RG-I segment and relatively rich in galactose, which may lead to the observed higher affinity for galectin-3 [78]. Gal-3 binding is thought to occur through the carbohydrate recognition domain (CRD) in the protein and can involve the N-terminal tail or a two-step interaction process. Pectins with diverse molecular structures can interact with Gal-3, including RG-I polysaccharides with long galactan side chains and unesterified pectins with non-substituted GalA segments [18]. The physicochemical changes from CP to MCP due to β-elimination increase pectin solubility and enrich “pharmacophores” found in the RG-I domain of pectin, which are galactans rich in terminal β-galactosides [79]. These galactans can be recognized by the CRD of Gal-3, the target of MCP in vivo models. The molecular mass plays a crucial role in MCP pharmacokinetics, affecting blood concentration, absorption, and excretion [79]. 

Another well-known benefit effect of MCP is its cytotoxicity, such as in breast cancer [80], prostate carcinoma metastasis [81], and gastric cancer [58], even though it is not an attribute exclusively of citrus pectin. In vivo studies have shown inhibition of tumor growth using heat- or chemically-modified pectins of sunflower [76], apple [82], artichoke [83], and lemon [28,74]. Sabater et al. [83] tested samples of modified pectin of artichoke without galactose and arabinose, interestingly observing that the absence of galactose decreased the anti-inflammatory effect, while the absence of arabinose did not change its anti-inflammatory properties. The inhibition of carcinogenesis happens through several mechanisms, including inflammatory suppression, inhibition of tumor survival signaling and induction of apoptosis, regulation of the cell cycle, suppression of inflammation, and reduction of tumor cell adhesion to endothelial cells, critical steps in metastasis [47]. Down-regulation of Gal-3 is also related to reducing tumor growth and increasing the sensitivity of tumor cells to chemotherapy drugs due to the protein’s involvement in apoptosis-resistance and drug-resistance pathways [79].

A study by Ren et al. [84] found that low molecular weight pectin from ginseng roots had a greater effect on weight loss and enhanced glucose and lipid metabolism in type 2 diabetic rats by inhibiting the expression of downstream lipid synthesis genes, mainly due to the action of RG-I fractions. Supplementing pectin on diets presented good results on cholesterol-lowering properties in hamsters, with a significant reduction in serum cholesterol levels compared to the control group. 5% DE pectin was able to downregulate genes related to lipid synthesis and upregulate genes related to lipid degradation [85]. Another study showed that pectin supplementation reduced cholesterol levels in hypercholesterolemic rabbits [86]. The increase in viscosity caused by pectins, due to their structural and chemical composition, is thought to reduce the diffusion and, consequently, the intestinal absorption of available carbohydrates [87]. In addition, pectin interacts with amyloglucosidase, avoiding the hydrolysis of starch into glucose [88].

Studies with lipopolysaccharide-challenged piglets showed that pectin supplementation improved gut health by alleviating morphological damage and restoring goblet cell numbers in the cecum. It also improved gut microbiota, increased beneficial bacteria, and improved the gut barrier and immunity by regulating cytokine expression through the attenuation of its receptor signaling [89,90]. Flies fed with low molecular weight pectin-enriched diets had a longer lifespan due to several mechanisms related to the pectin effect, such as decreasing reactive oxygen species (ROS), modulating gut microbiota and homeostasis, and regulating the expression of genes related to autophagy [91]. The action of pectin on gut microbiota was also observed as a booster factor in enhancing the efficacy of immunotherapy for colorectal cancer (CRC), as the fermentation product SCFA butyrate assisted T cell infiltration in tumors of mice [92]. Another application of pectin is demonstrated by Yuan et al. [93], whose goal was to build a nanotube system to carry a drug against *Salmonella* to the intestine. Low-methoxy pectin was incorporated as a protective film to ensure that the nanotube would pass through the gastric acid unharmed and reach the intestine in a nondegraded form. In vivo assays revealed that not only was the pectic film effective as a protection film, but it also played a role by attenuating enteritis caused by *Salmonella* and modulating homeostasis and the microbiota balance. 

These studies have typically used animal models, such as rats or mice, to evaluate the effects of native and modified pectin. They provide valuable information about the molecular and physiological systems of treatments and how these potential therapies may affect the disease process. Clinical trials, on the other hand, are important because they are the only way to test the safety and efficacy of a given therapy in humans. They provide real-world data on how a given therapy influences the health and well-being of individuals and can help determine the best way to use a given therapy in the clinic. Therefore, the complementarity of these two methods is essential for advancing our understanding of the biology of a disease and the development of safe and effective therapies using plant-derived compounds.

## 5. As Close as It Gets: Clinical and Preclinical Advances to Indicate the Best Plant-Derived Pectin

There is a great deal of interest in the impact of plant-derived dietary fiber on health and disease [94]. As mentioned, beneficial effects could be direct and/or indirect, such as the stimulation of the gastrointestinal immune barrier and the benefits of protecting against cardiovascular diseases [17]. 

In 2019, a meta-analysis compiling 58 clinical trials showed that a higher fiber intake (between 25 g/day and 29 g/day) was associated with reduced body weight, systolic blood pressure, and total cholesterol when comparing groups with low or high fiber intake [95]. 

Pectins’ benefits come from their composition and the presence of specific structural domains, which have bioactive properties. Pectin’s ability to form a viscous gel and the binding of cholesterol and bile acids, for example, promote their excretion and reduce absorption. High-DM pectins are known to lower plasma cholesterol owing to their lesser solubility [41,96].

The study carried out by Kumar and Chauhan [97] demonstrated that the formation of a lipase–pectin complex results in lipase inhibition. As it is a weak acid, pectin resists dissociation in the gastric environment and thereby binds covalently to the active sites of pancreatic lipase. The hypothesis is that the single-bond CO_2_H groups protonate histidine, and the single-bond OH group of the serine-histidine-aspartic/glutamic acid triad of lipase is what stops the mechanisms to which lipase action is subservient [41,96].

**Figure 3:**
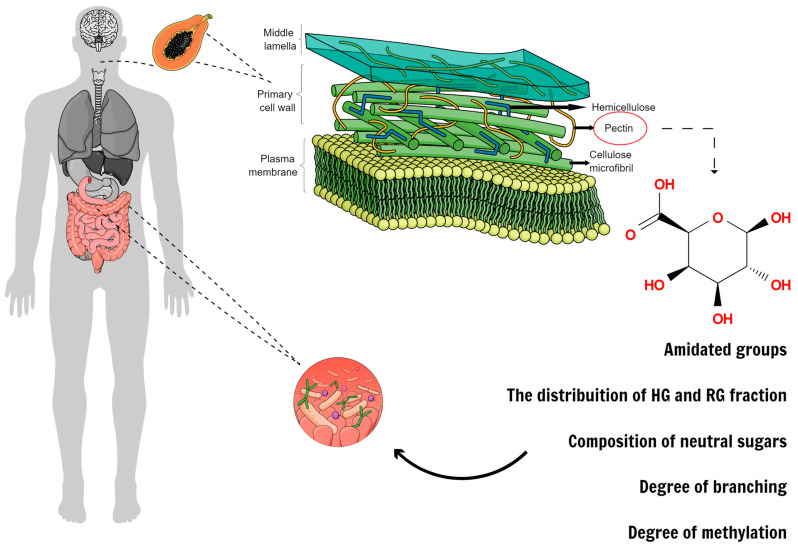
Pectins are primary cell wall components in fruits and vegetables, and depending on their structure, they can be more or less fermented or recognized by different receptors.

Since pectin’s chemical structure is essential to confer benefits, molecular modifications began to be studied as well. Pectin modification predisposes smaller fragments of the pectin molecule during intestinal fermentation, which enhances metabolite production (Figure 3). These fragments can also bind to Gal-3 and block its activity, impairing tumor progression. This finding is supported by data from various settings, including in vitro and in vivo studies and human clinical trials [51]. However, inhibition power has been contested by some study groups due to molecular size and spatial conformation, which have been taken into account in a previous review [18].

A summary of different pectin applications in cancer and other diseases is available in Table 3. Clinical trials with fewer than 20 volunteers (*n* < 20) were excluded from the summary due to standardization. Small studies and case reports have some disadvantages, but they can be useful in identifying rare clinical findings and generating hypotheses for further research.

In this context, four studies can be cited from the same research group that used commercially modified pectin (MCP) to investigate the potential for the elimination of heavy metals through urine. This specific MCP has been characterized in an earlier in vitro study by one of the same authors as having a smaller size than 15 kDa, under 5% DE, and a content of approximately 10% RG-II. According to the authors of the study, modification is believed to enable preferential transport of short-chain galactans and AG from the small intestinal epithelium into the circulation [98,99,100,101]. 

**Table 3:** Clinical trials with pectins and/or MCP.

Clinical Hypothesis	Type of Pectin Used	Characteristics of Pectin Used	Study Design	Study Population	N° of Participants	Summary of Results
**Direct inhibition of Gal-3 may reduce subclinical cardiac fibrosis**	MCP (PectaSol) by EcoNugenics, Inc.	Low molecular weight, DE under 5%, approximately 10% RG-II and enzymatic modification,	Randomized placebo-controlled	USA	52	Demonstrated a higher baseline Gal-3 levels in women compared with men, validated previous associations of Gal-3 with clinical factors. Did not influence surrogate measures of cardiac fibrosis, including echocardiographic [102]
**Evaluate the effect of MCP in men with prostate cancer**	MCP (PectaSol) by EcoNugenics, Inc.	Low molecular weight, DE under 5%, approximately 10% RG-II and enzymatic modification,	Prospective phase II study	USA	59	The results suggested that MCP in BRPC has a potential benefit, as evidenced by changes in prostate-specific antigen doubling time (PSADT) and lower-than-expected rates of disease progression compared to historical data. The exceptionally low incidence of toxicities was a promising result found as well [103].
**Bananas and pectin seem to be potential therapeutic agents for diarrhea**	Green banana and pectin (Sigma, St. Louis, MO, USA)	Not applicable	Double-blind controlled	Bangladesh	62	Almost all children in the banana and pectin group had recovered from clinical diarrhea. Pectin is at least as effective as banana or has slightly better effects on stool volume and quality, although more expensive [104].
**The cholesterol-lowering properties of the viscous fiber pectin may depend on its physicochemical Properties**	Commercial and experimental pectins (PEC1 and PEC2) *	PEC1 corresponded to 15 g/day of cellulose (control). PEC2 only corresponded to capsules that were reduced to 0.325 g of pectin if compared with PEC1.	Randomized crossover	The Netherlands	60	Pectin source and type affect cholesterol lowering. The efficacy of pectin to reduce cholesterol was dependent on the physicochemical properties and improved with MW (molecular weight) and DE (degree of esterification) [105].
**To examine and verify the clinical effects of POF**	Pectin-containing oligomeric formula (POF)	Pectin content, 0.9 g.	Randomized	Japan	198	POF is less likely to cause enteral nutrition (EN) related events, such as diarrhea, than standard polymeric formula (SPF) [106].
**To investigate the effects of pectins supplementation on gastrointestinal barrier function**	GENU^®^ BETA pectin	Free-flowing powder, Particle size: Less than 1% gum on a 0.250 mm test sieve and DE typically 55%	Randomized, double-blind, placebo-controlled	The Netherlands	97	With 4 weeks of intervention, intestinal barrier function was not affected by pectin supplementation for both groups (healthy adults and elderly people) [107].
**MCP could control cancer growth and tumor metastasis**	MCP	DE below 20%, pH modified by partial neutralization as Potassium/Sodium salt.	Pilot trial	Germany	49	The treatment with MCP did not induce antitumoral responses. However, a 50% decrease in the serum PSA level after 16 weeks of treatment, but more studies will be necessary [108].

* PEC1 corresponded to 15 g/day of cellulose (control) or different types of pectin that were incorporated into cereal bars, fruit preparations, and capsules. PEC2 only corresponded to capsules that were reduced to 0.325 g of pectin if compared with PEC1. The commercial and experimental pectins used in PEC1 and PEC2 were characterized by standard analytical methods, according to JECFA (2009).

Pectin modified with chemicals, heating, radiation, and enzymes all have deeper antitumor activity than unmodified pectin, but most clinical trials do not emphasize the structural characteristics of pectin. In the current review, we strongly emphasize that a physicochemical characterization must be performed to specify from which plant source the biologically active pectin must be extracted and modified. Studies suggest that the antitumor mechanisms of MCP are correlated with its anti-apoptotic activity and that MCP acts by sensitizing tumor cells to chemotherapeutic drugs. Nevertheless, it is necessary to clarify this relationship and characterize the structures that induce apoptosis [41].

In the context of pharmacokinetics, pectin has also been used as a drug delivery system (DDS) because of its biocompatibility, health advantages, nontoxicity, biodegradability, low production cost, and wide availability. However, pectin’s rich hydrophilic functional groups, including the hydroxyl, free carboxyl, and methyl ester groups, and its formulations have the potential to expand under physiological conditions, resulting in premature drug release. As a result, researchers have attempted to alter the structure of pectin; an example is the polymer nanoparticles that are being studied [109].

Two studies investigated the use of pectins as a pharmacological vehicle in nasal sprays for the management of breakthrough cancer pain (BTPC). In the short-term study with Fentanyl Pectin Nasal Spray (FPNS), gel formation was the technological target for pectin inclusion. This formation occurs due to the interaction of the low-methoxyl (LM) pectin with calcium ions present in the mucosal fluid, which allows locally acting drugs to reside for longer at the site of application. The treatment was efficacious, safe, and well tolerated for the treatment of BTPC, showing a vehicle application for this polysaccharide [110,111,112].

## 6. Trending Technologies for Biomedical Applications

As briefly commented in the introduction, different pectin technologies aimed at human health have been trending in recent years. Considering the pectin’s physicochemical characteristics and the fact that the gastrointestinal tract does not digest it, one of the approaches studied is the use of pectin as a nanoparticle component to encapsulate materials to enhance their properties or bioavailability. This has intrinsic importance for the structural characteristics of the pectin molecule, such as the net charge from the HG backbone, the interaction between side chains, and hydrogel properties. These nanoparticles can be formed by merging other macromolecules, such as proteins or other carbohydrates, to enhance the stabilization of the structure formed [25,113]. 

One emerging example is the nanoencapsulation of anthocyanin extracted from blackberries, which is a natural pigment with antioxidant effects and biological properties, acting on modulation of inflammation that can have a lower bioavailability due to gastric, microbiota, and intestinal modifications. The nanoparticles were created with pectin and lysozyme [114]. The pectin-lysozyme complex was also used for β-lactose encapsulation, an anomeric form of lactose with some pharmacological potential [11].

Whereas the association between a protein and pectin creates a nanoparticle, there are also solid-lipid nanoparticles (SLN). This was made with pectin and skimmed milk powder, an emulsifying agent, to encapsulate curcumin, a lipophilic antioxidant polyphenol with health-promoting properties, including cytotoxic activities [12]. Controlled release is one of the encapsulation goals shown in sulfasalazine-loaded amidated pectin microparticles through Eudragit S100 (a pH-sensitive polymer) coated capsules for delivery of drugs dependent on pH and time [13]. New technologies using pectin are not limited to nanoparticles; it can be used in the formulation of mini tablets, such as those containing 5-Fluorouracil, Eudragit S100, guar gum, pectin (colon-specific polysaccharides), magnesium stearate, and crushed powder for co-administration of prebiotics and drugs for the treatment of colon cancer [14].

Considering that pectin has the property to form gels, and that in vitro studies with cancer cells can utilize hydrogels in the cell culture, new technology can be tested on in vitro tumor models containing spheroid cell cultures [115]. A 3D environment is an approach that can provide the complexity and heterogeneity of human tumors that are dependent on environmental signals and the extracellular matrix (ECM) [116]. Thus, to develop an ECM-based model to provide an environment with the same stiffness as the tumor in vitro, thermo-responsive hybrid hydrogels in chitosan-pectin are suitable for cell encapsulation because of the presence of components similar to the ECM composition. To create this hybrid hydrogel, pectin was mixed with chitosan, and after chitosan gelation, a stable semi-interpenetrating polymer network formed, permeable to molecules with molecular sizes between 3 and 70 kDa [115]. Thereby, these chitosan-pectin hydrogels may be helpful in standardizing the in vitro culture of 3D tumor models for tumor progression and studies of drug response.

## 7. Final Thoughts

Pectins and other types of dietary fiber provide diverse health benefits and good assistance in the treatment of different diseases, as well as maintaining a healthier gastrointestinal environment. Despite several studies in rodent models showing that varied types of fiber supplementation have a large effect on the composition of the gut microbiota and the production of metabolites such as SCFAs, data in humans are more limited, and the revealed effects are relatively modest [94]. This only highlights the need to expand and further explore the activity and application of those polysaccharides in the human diet and, in the future, pharmacological approaches. The present review fulfills its purpose by covering a literature gap by thoroughly evaluating pectin at all levels of clinical research. It includes examining its in vitro properties, especially the cancer-cytotoxic properties, the impact on gut health through fermentation byproducts, and the immunomodulation of specific receptors. The review also explores in vivo models and looks toward human testing with varied outcomes to determine if the observed effects are somehow translated to human health. Most of all, this review opens space for increasing focus in technological research focusing on pectin applications as molecules of interest regarding effects and stabilizing and carrying properties. However, the review still has its limitations, and therefore additional approaches using multi-ranging study models with a focus on non-included pathologies such as inflammatory bowel disease would be highly beneficial to the field.

## Figures and Tables

**Table 1:** Summary of different plant-derived pectins, the extraction/modification process to obtain them, and respective resulting structures.

Plant Material	Extraction/Modification Procedure	Resulted Structure	Reference
Tomato plants	Only water (WSP); different concentration imidazole (ISP); sodium carbonate and borohydride (DASP); potassium hydroxide (KOH)	WSP, ISP, and DASP all consisted of pectins, with DASP having RGI fragments; the KOH fraction was composed of xyloglucans, arabinoxylans, and galactans	[32]
Banana peel	Acetic, citric, hydrochloric (HCl), and nitric (HNO_3_) acids at pH 2 or 3	Acetic, hydrochloric, and nitric acid resulted in LMPs with shorter chains, while citric acid resulted in HMs with longer chains at both pHs	[34]
Pea hulls	HNO_3_ or citric acid, under variable pH, temperature, and time conditions	DM ranged between 36 to 49%, with no differences between acids; higher pH (2) yielded more neutral sugars and less uronic acids, proportionally, for both acids	[36]
Commercial citrus pectin with 73% DM	De-esterification with variable NaOH concentrations, with hydrochloric acid pH adjustment	Besides lower DM, de-esterified pectin had lower molecular weights, zeta-potential, polydispersity index (PDI), and HG proportion while having higher RGI	[37]
Fuji apple-peel waste	Tartaric, malic, and citric acids, at 0.1 or 1.0 M	Malic and citric acids yielded higher DM pectins than tartaric (65–67% compared to 59%); higher concentrations seem to indicate a tendency of reduced DMs; Mw varied from 26 to 110 kDa	[38]
Dried apple-pomace	H_2_SO_4_, or endo-β-1,4-xylanase (Xyl) and endo-cellulase (Cel)	Acid-treated had the lowest Mw, GalA content, and DM; Cel + Xyl treatment resulted in a close Mw, with much higher GalA (74.7% × 59.9%) and DM (67.5 × 56.1%); Acid treatment also led to higher Glc and Ara, while Cel + Xyl presented more Gal, Rha, and Man	[39]
Raspberry	HCl or enzymatic hydrolysis (α-amylase, papain, cellulose), following step-wise alcohol precipitation	HCl-modified pectins had much lower Mw, PDI, and Xyl, with absent Man residues, while also having higher amounts of Gal and GalA. Other neutral sugars, such as Rha and Glu, were also higher in enzyme-extracted pectin	[40]
Commercial apple pectin (74%)	Ultrasound with amplitudes from 20 to 60% for 60 min at 25 °C and temperatures ranging from 5 to 45 °C for 30% sonication amplitude	Temperature variation seemed to decrease the Mw reduction (299 kDa × 361 kDa, comparing 5 to 45 °C); the lowest Mw and PDI values were obtained at 60 min; DM and RGI proportion was reduced by ultrasound intensity and time	[42]
Grapefruit peel	50% Ultrasound amplitude in 2-s cycles (UP), with sporadic agitation; other fraction stood 90 min under 80 °C without ultrasound (CP)	Mw was slightly lower for the UP (109.54 × 132.01 kDa), as well as GalA, Glc, and Man residues; DM, as well as Rha, Gal, and Xyl residues, were comparable; Ara was much higher in UP (12.72 × 4.43 mol%)	[43]
Citrus peel	NaHCO_3_ in different concentrations and 30% H_2_O_2_ in water with ultrasound; compared to the same medium without ultrasound	The chemical + ultrasound group showed a 30-fold Mw reduction (33 × 1088 kDa), and the chemical group a 10-fold reduction (103 kDa); every monosaccharide proportion was comparable, besides a higher proportion of Fuc (1.81 × 0.52 mol%) and a lower GalA proportion (54.39 × 57.23 mol%) between groups; both had higher Gal contents than control	[44]
Commercial citrus pectin	Chemical modification through saponification with NaOH, acidification to pH 3.0 with HCl, and neutralization with NaOH (MCP); high temperature through heating in an autoclave for 121 °C for 30 min two times, on two different days (HTCP)	Acid treatment removed completely all methylations; the Mw of both were comparable (61 and 9 kDa peaks for MCP; 24 kDa for HTCP); all pectins had the same monosaccharide profile, mostly composed by GalA, with Rha, Gal, and Ara as secondary components	[45]
Commercial apple pectin	Solution pH was adjusted to H_2_SO_4_, and the solution was incubated in a microfluidizer; optimal variables were 155 MPa and 6 cycles	Almost 33% of the pectin content was converted into pectic oligosaccharides (POS); GalA content was greatly decreased in POS (29.56 × 71.68 wt%); Ara, Gal, Rha, Glc, and Xyl had a 3-fold increase in the POS sample	[46]

**Table 2:** Summary of in vitro effects from pectin poly- and oligosaccharides from varied sources.

Pectin/Fragment Used	In Vitro Model	Main Results Found	Authors
Modified sugar beet pectin fragments	HT29 and DLD1 cell culture	Pectin stimulated apoptosis and detachment of HT29 cells; the galactan fragment was most efficient in lowering cell proliferation	[48]
Size-fractioned oligo- and polysaccharides from MCP	HCT116, HT29, and PC3 cell lines	MCP30/10 and 10/3 stimulated cytotoxicity through necroptosis and necrosis on the HCT116 cell line; smaller fragments significantly reduced homotypic aggregation and cell migration	[49]
Papaya pectin from different ripening points	HCT116, HT29, and PC3 cell line	Pectins extracted from the third day after harvesting inhibited cancer cell aggregation, migration, and further reduced viability while enhancing cytotoxicity; they also increased pAkt, pErk1/2, p21, and caspase-3 protein expression	[52]
Ultrasound-fragmented Sweet Potato pectin with high GalA content and low DM	Oxygen radical absorbance capacity and Ferric reducing antioxidant power assay (ORAC and FRAP)	The most fragmented pectin performed better in both antioxidant assays, starting in lower doses, compared to the other pectins	[53]
Enzymatically extracted apple pectin	HCT116 and Caco-2 cell culture	Pectins were able to synergize with Irinotecan increasing colorectal cancer cell cytotoxicity through apoptosis induction compared to drug-only controls; ROS levels increased; reduced IL-6 and COX-2 in LPS-induced HCT116 cells; pectins also reduced *E. coli* adherence to colorectal cancer cells	[54]
Acid and heat-treated pectic extracts from olives	Caco-2 and THP-1 cell culture	Higher doses (3.33 and 10 mg/mL) were able to cease cell proliferation completely up to seven days; pectin extracts successfully induced caspase-3 activity, compared to MCP and control	[55]
Varied potato pectins, citrus polygalacturonic acid, larch AG	HT29, DLD1, HCT116, LoVo; and Caco-2 cell culture	Potato RGI was the most effective in reducing cell proliferation in a dose-response manner; authors detected ICAM-1 downregulation and proposed reduction of proliferation was due to cell detachment	[56]
Low-molecular-weight citrus pectin	AGS and SW-480 cell culture	LCP lowered cell viability, proliferation, and cell cycle progression; the pectin had comparable values of Cyclin B1 and Bcl-xL downregulation to 5-FU	[58]
Papaya pectin from different ripening points	HEK-TLR reporter cell lines	The ripe-fruits pectins activated TLR3, 5, and 9; all pectin samples activated TLR2 and 4; only unripe-fruit pectins inhibited TLR3 and TLR9	[66]
Acid and neutral fractions of papaya pectins from different ripening points	HCT116 and HT29 cell culture, recombinant human Gal-3 hemagglutination	The acid fraction from four days after harvest was able to inhibit Gal-3 hemagglutination from the second-lowest dose onward; cell viability was reduced mostly by full water-soluble fractions or acid fractions	[72]
Oligosaccharides from jaboticaba, plum, and papaya flours	HCT116 cell culture and recombinant human Gal-3 hemagglutination	Only jaboticaba oligosaccharides were able to inhibit Gal-3 hemagglutination and colorectal cancer cell viability	[73]
Lemon pectins with varying DMs	HEK-TLR reporter cell lines	Low-DM pectins inhibited TLR2 activity way more than higher-DM ones; specific TLR2-TLR1 heterodimer inhibition was observed	[74]
Lemon pectins with varying DMs	Human pancreatic islets and MIN6 cells	Low-DM pectins showed protective effects on human islets against streptozotocin and IL1b + IFN-y + TNF-a inflammatory stress through a Gal-3 binding-dependent way	[75]
High-temperature sunflower pectin and polygalacturonic acid	CT26 colorectal cancer cell line	Pectins were able to upregulate JNK, ERK, and p38 phosphorylation while down-regulating Akt time-dependently; dose-dependent stimulation of apoptosis was also observed in vitro	[76]

## Data Availability

No new data were created or analyzed in this study. Data sharing is not applicable to this article.
